# To Buy or Not to Buy? A Research on the Relationship Between Traceable Food Extrinsic Cues and Consumers’ Purchase Intention

**DOI:** 10.3389/fpsyg.2022.873941

**Published:** 2022-04-25

**Authors:** Li Ge

**Affiliations:** School of Computer Science and Technology, Weinan Normal University, Weinan, China

**Keywords:** traceable certification credibility, purchase intention, traceability knowledge, traceable information quality, peer influence

## Abstract

With the prevalence of traceability technology in the turbulent Internet age, traceable food has become an important tool in addressing food safety issues. Under the combined effect of frequent food safety problems and sustainable development of traceability industry, the research on traceable food consumer behavior has become more extensive. However, it is still not fully understood how the multiple information brought by traceability affects consumers’ purchase decision. This study proposes the effects of traceability knowledge, traceable information quality and traceable certification credibility on traceable food purchase intention *via* the mediation of perceived risk and perceived value, and integrates the moderating effect of peer influence in the context of Internet age into a research framework. The analytical results indicate that traceability knowledge, traceable information quality, and traceability certification credibility indirectly affect consumers’ traceable food purchase intention through perceived risk and perceived value, while traceability knowledge, perceived risk, and perceived value directly affect “traceable food purchase intention.” Furthermore, peer influence was found to be a significant moderator in the relationship between perceived risk (perceived value) and “traceable food purchase intention.” Finally, based on the research results, traceability companies are suggested to focus on cultivating the traceable consumption habits. Meanwhile, although traceable food quality is the top priority, companies should also attach importance to the communication and interaction with consumer.

## Introduction

In the turbulent Internet age, “consumer is increasingly inseparable from modern information technology, while food traceability is receiving more and more attention as a means to ensure food safety. Food safety issues have become a core factor in consumers’ food purchase decision” (Yue et al., 2017). In December 2019, the U.S. Food and Drug Administration notified Almark Foods “of their hard-boiled and peeled eggs in pails manufactured at the Gainesville facility may be associated with a Listeria monocytogenes outbreak, which has been linked to several reported illnesses and one reported death” (SJTU Food Safety Research Center, 2019). In 2008, more than 50,000 Chinese children had been reported to have kidney stones associated with melamine contamination of dairy products. The incident hit the reputation of China-made products hard, and several countries banned the import of Chinese dairy products. Due to the specific product and social attributes of food, consumers are unable to observe the production process, resulting in asymmetry in food safety information attributes (Hobbs, 2004). The imperfection of Chinese credit system exacerbates consumers’ concerns about the lack of confidence in food quality and increases uncertainty in their purchasing process.

With the application of modern information technology, food traceability systems provide new ideas to address information asymmetry in food consumption process. As a main tool to eliminate information asymmetry and prevent food safety risks, it is considered to be of considerable importance (Liu et al., 2018). By increasing the transparency of food information, the traceability ensures the quality of food and reduces the concerns about food safety (Badia-Melis et al., 2015), thus enhancing consumers’ trust (Rijswijk et al., 2008).

As an important promoter and main participant in food safety traceability system, paying attention to consumers’ perception of traceable food has a catalytic role in improving the construction of traceability system. At the same time, studying the extrinsic cues of traceable food has great implications for discovering consumers’ purchase intention mechanism. Therefore, this article constructs a traceable food purchase intention (TFPI) model from the interaction perspective between extrinsic cues of traceable food and consumers’ cognitive evaluation to empirically demonstrate the relationships among traceability knowledge (TK), traceable certification credibility (TCC), traceable information quality (TIQ), perceived risk (PR), perceived value (PV), and purchase intention. It provides a reference basis for identifying the key elements of consumers’ purchase decision and provides theoretical and academic support for the promotion of traceable food. Furthermore, in the turbulent Internet age, peers have a more shaping and reinforcing effect on individuals compared to other groups (Schmidt and Tyler, 1975); the more information consumers received from peers, the stronger their motivation to conform to peers’ expectations. This article explores the moderating effect of peer influence (PI) between consumers’ cognitive evaluation and consumers’ purchase decision.

## Background and Hypotheses Development

Cox (1962) defined cues as product-related information, e.g., product color, shape, price, seller’s attitude, and friend’s opinion. Olson and Jacoby (1972) refined cues into intrinsic cues and extrinsic cues. They argued that in purchase decision consumers make judgments based on either the self attributes of the product (intrinsic cues) or non-self attributes (extrinsic cues). Cue-based decision-making is considered an independent consumer decision model (Hamlin, 2010). Specifically for traceable food, its intrinsic cues include physical cues such as color, shape, taste, and appearance of food (Verbeke and Ward, 2006), while numerous studies have shown that origin, price, production information, quality assurance, label composition, and third-party certification (Rijswijk et al., 2008; Wang and Tsai, 2019) are important extrinsic cues with benefits of food safety, health, nature, quality, trust, control assurance, and environmental protection.

Starting from consumers’ subjective feeling, the cognitivist approach is concerned with consumers’ decision and behavior. According to cognitivism, the organism influences its relationship with individuals’ response by means of cognition and evaluation after being stimulated (Arnold, 2010). Research related to consumers’ cognitive evaluation varies with different research dimensions. On the whole, the empirical studies based on consumers’ cognitive evaluation can be broadly classified into two categories. One is emotional studies, such as trust (Han and Kim, 2016), satisfaction (Hsu et al., 2012), and familiarity (Das, 2015). This category of research considers consumers’ cognitive evaluation as a result of changes in individuals’ emotion caused by extrinsic stimuli, which lead to different behavioral responses. The other is cognitive studies, such as perceived informativeness (Liu et al., 2019), PR (Yuan et al., 2020), and PV (Jayashankar et al., 2018). This category of research considers cognitive evaluation as consumers’ perceptions brought by extrinsic stimuli, and a comprehensive evaluation of these perceptions will further influence consumers’ subsequent behavior. Richardson et al. (1994) explored the relationship between consumers’ perception of quality and intrinsic/extrinsic cues. The result showed that both extrinsic and intrinsic cues have an impact on consumers’ perceived quality, but extrinsic cues played a more important role in the process. Traceable food is traced throughout the whole life cycle by technologies such as one-thing-one-code and Internet of Things. Due to this distinguishing characteristic, consumers rely more on extrinsic cues provided by traceability in their purchase decision-making. Bernués et al. (2003) proposed a product quality perception model. He argued that product quality consists of objective quality and subjective quality. The objective quality of a product is the value, intrinsic and extrinsic characteristics of a product during production, supply and distribution, while the subjective quality is the perception of search, credence, and experience attributes of the objective quality. According to this model, the extrinsic cues influencing consumers’ traceable food purchase can be divided into search, credence, and experience cues. Consumers will draw their purchase conclusions from the comprehensive evaluation of these cues. This article proposes that the TK acquired by consumer, the perceived information quality carried by the traceability labels, and the credibility of traceability certification brought by the certification marks are the most important experience, search, and credence attributes, which represents consumers’ overall quality perception of traceable food.

### Traceability Knowledge

In the studies of consumer behavior, knowledge is considered to have an influence on all stages of decision-making process (Peng and Chen, 2019), particularly on how consumers assess the inherent value or risk of the product and their purchase behavior (Yuan et al., 2020). Buaprommee and Polyorat (2016) found that as an important cognitive factor influencing consumers’ purchasing behavior, consumers’ knowledge of a product enabled them to better understand the product and make sound purchase decisions. Klerck and Sweeney (2010) argued that consumers’ TK has an important role in elucidating consumer behavior, especially in terms of information search and processing. Consumers’ knowledge of nutrition, health, and traceability can facilitate their use of traceability information as the benefits offered by traceable foods can be easily explained and identified. Therefore, TK has an impact on food information collection, attitudes, and purchase intention.

Lin et al. (2017) have suggested that consumers’ green knowledge contributes positively to promoting consumers’ green PV in the study of the effect of green brand innovation on brand loyalty. Wang and Hazen (2015) empirically verified that consumers’ PV and PR are influenced by consumers’ product knowledge, and suggested product knowledge as one of the most important influencing factors on consumers’ purchase behavior. Similarly, food-related knowledge stimulates consumers’ purchase intention (McClure and Seock, 2020). Sharaf and Isa (2017) have noted that knowledge significantly affects consumers’ green purchase intention in his study of students’ green consumption behavior. Paul et al. (2016) noted that environmental knowledge positively influenced consumers’ purchase intention in their study of green product consumption intentions. Suki (2016) also demonstrated this hypothesis in the study of green product uses. Thus, the following three hypotheses were proposed:

**Hypothesis 1a**. TK has a significant negative effect on PR.

**Hypothesis 1b**. TK has a significant positive effect on PV.

**Hypothesis 1c**. TK has a significant positive effect on TFPI.

### Traceable Information Quality

When consumers are aware of the risks associated with food safety, they are motivated to seek more information through the use of traceability system (Yoo et al., 2015). Food traceability system allows consumers to have a more transparent understanding of the entire process of food products from production to distribution, thus becoming an effective way for users to avoid safety risks (Liu and Niyongira, 2017). Wang et al. (2017) found that traceability system provides consumers with timely information to assess and enhance their experience, thus helping companies to improve consumers’ perceptions of product reliability.

Cheung et al. (2008) argued that information quality perceived by consumers determined their purchase decision of goods or services. They also state that consumers’ perception of information quality is an important factor in evaluating their potential purchase behavior. Mun et al. (2013) stated that perceived information quality affects PR. Zheng et al. (2017) have noted that there is a positive relationship between information quality and PV. Furthermore, Menozzi et al. (2015) noted that food traceability information, especially information about pest control and intermediate product inputs, was effective in improving consumer purchase intention. Riccioli et al. (2019) identified traceability information as the primary aspect that positively influenced consumers’ purchase intention compared to other quality certifications. Based on the above discussion, the following three hypotheses emerged:

**Hypothesis 2a**. TIQ has a significant negative effect on PR.

**Hypothesis 2b**. TIQ has a significant positive effect on PV.

**Hypothesis 2c**. TIQ has a significant positive effect on TFPI.

### Traceable Certification Credibility

TCC represents the degree of trust consumers found in the certification declaration provided by the government or a third-party organization. As a credible form of certification, food safety certification is considered an important means to judge the level of food safety, convey trust within the industry, and have a significant impact on consumers’ decisions (Brach et al., 2017). Purchasing traceable food through a reputable third-party certifier ensures quality (Giacomarra et al., 2016) and credibility (Brach et al., 2017), alleviating consumers’ information asymmetry. In turn, companies can also gain more benefits from certification. As a fundamental element of industry competition, companies seek continuous quality improvement through certification to attract more customers (Bai et al., 2013).

Consumers’ trust in the product is influenced by the credibility of the certification body (Bai et al., 2013). When studying the credibility of sources, Filieri (2015) found that the credibility of the source enhanced trust in the information provided by the website and thus influenced consumers’ judgment. In a study of Chinese consumers’ purchase intention, Wu et al. (2015) found that certification was the most important traceable food purchase characteristic, followed by appearance and traceability information. Cui et al. (2019) found out that the credibility of food safety information has a significant effect on consumers’ PR. It has also been shown that third-party certification can provide informational cues and help reduce consumers’ perceptions of product risk (Brach et al., 2017). Meanwhile, Lin et al. (2017) proved that the credibility of ads was positively related to consumers’ PV of location-based mobile ads. Wu et al. (2016) found that consumers’ perception of the credibility of third-party certified traceable information significantly influenced consumers’ purchase intention. Kim and Song (2020) noted that the trust in certified traceable food significantly influences consumers’ purchase intention in their study of certified label traceability. Therefore, the following three hypotheses were proposed:

**Hypothesis 3a**. TCC has a significant negative effect on PR.

**Hypothesis 3b**. TCC has a significant positive effect on PV.

**Hypothesis 3c**. TCC has a significant positive effect on TFPI.

### Cognitive Evaluation

The study of cognitive-emotional organism factors in cognitive evaluation theory suggests that consumers’ psychological responses to extrinsic cues are a process in which individuals move from primary intuitive cognition, to emotional transformation, and then to higher-level cognition (Kim and Moon, 2009). Therefore, this article adopted consumers’ high-level cognition (evaluation and judgment) as the final psychological judgment of consumers’ cognitive evaluation of traceable food. Ueland et al. (2012) proposed a benefit-risk assessment mode, suggesting that consumers’ evaluation is composed of perceived benefits and PR. Specific to traceable food, the price and premium are also an indispensable consideration in the process of consumers’ evaluation and comparison; meanwhile, PV theory (Zeithaml, 1988) holds that consumers’ PV is jointly determined by their perceived benefits and the price they pay. Therefore, this article considers that consumers’ evaluation and judgment of traceable food is composed of PR and PV.

Tzavlopoulos et al. (2019) proposed that PR is negatively related to PV in their study of e-commerce quality. Liang et al. (2015) concluded that consumers’ PR significantly affects consumers’ PV. Abror et al. (2021) have found out that PR has a significant impact on PV in their study of relationship between PR and tourists’ trust. For many people, traceable food is a new experience that differentiates from traditional agri-food. Therefore, greater uncertainty may result in a greater degree of PR, which in turn may affect changes in consumers’ PV.

PR theory suggests that consumers tend to minimize PR rather than maximize their purchase utility (Mitchell, 1999). That is, a decrease in PR leads to an increase in consumers’ willingness to purchase, thus PR negatively affects consumers’ purchase intention. In the study of relationship between cross-border E-tailer’s return policy and consumers’ purchase intention, Shao et al. (2021) have proposed that PR has a negative effect on the consumers’ purchase intention. Tran (2020) has also found that product risk influenced purchase intentions for online shopping. Zeithaml (1988) has stated that PV is one of the most important factors influencing purchase intention. Sweeney et al. (1999) have proposed that PV can build positive word-of-mouth effects and increase purchase intentions. Petraviiūt et al. (2021) have proposed that PV has positive effect on purchase intention of luxury brand. Therefore, the following hypotheses were proposed:

**Hypothesis 4**. PR has a significant negative effect on PV.

**Hypothesis 5**. PR has a significant negative effect on TFPI.

**Hypothesis 6**. PV has a significant positive effect on TFPI.

### Mediating Effect of Cognitive Evaluation

According to the cognitive evaluation theory, the internal state of the individual is regulated between external stimuli and behavioral responses (Arnold, 2010). That is, consumers need to evaluate the quality, price, and service of a product before purchase in order to make a more favorable decision. Previous research findings provided the theoretical basis for the hypothesis of the mediating influence of cognitive evaluation on the relationship between extrinsic cues of traceable food and consumers’ purchase intention. For example, Ponte et al. (2015) empirically examined the mediating role of PV between trust and consumers’ purchase intention. Liang et al. (2015) found that consumers’ online travel purchase intention was mediated by PV and PR. In this article, PR and PV were developed to measure the positive and negative effects of extrinsic stimuli on individuals’ perception and evaluation, respectively. Specifically, consumers’ perception of TK, TIQ, and TCC will influence consumers’ purchase intention through the mediating effects of PR and PV. Therefore, the following hypotheses were proposed:

**Hypothesis 7a**. PR plays a mediating role between TK and TFPI, and TK promotes the purchase intention by reducing PR.

**Hypothesis 7b**. PR plays a mediating role between TIQ and TFPI, and TIQ promotes purchase intention by reducing PR.

**Hypothesis 7c**. PR plays a mediating role between TCC and TFPI, and TCC promotes the purchase intention by reducing PR.

**Hypothesis 8a**. PV plays a mediating role between TK and TFPI, and TK promotes purchase intention by increasing PV.

**Hypothesis 8b**. PV plays a mediating role between TIQ and TFPI, and TIQ promotes purchase intention by increasing PV.

**Hypothesis 8c**. PV plays a mediating role between TCC and TFPI, and TCC promotes the purchase intention by increasing PV.

### Moderating Effect of Peer Influence

Hallinan and Williams (1990) concluded that peers as groups with similar social identities, geographical proximity, or similar individual characteristics are more easily understood by each other due to their similar individual characteristics (age) or psychological characteristics (interests, hobbies). Campbell and Alexander (1965) concluded PI as a phenomenon in which the behaviors and attitudes of individuals in a group are influenced by other individuals, leading to a convergence of behaviors and attitudes within the group. Rogers and Wheeler (1976) explained the mechanism of PI in the social network, suggesting that interpersonal communication is more likely to occur among peers because of similar psychological traits, or interests, which makes it easier to understand and trust each other and thus achieve effective communication. Kandel (1985) explained the mechanism of PI from a social psychological perspective. He suggested that due to the similarity of certain characteristics, individuals engaged in like-matching and interaction; during the process, they are implicitly influenced, which in turn leads to convergence of behavior or views. As “social beings,” individuals are susceptible to peer interactions in order to better integrate into their groups and achieve a better social status. PI improves individuals’ attitudes, knowledge, and behavior by internalizing and assimilating information provided by peers through imitative learning mechanisms, resulting in convergence of views or behaviors among individuals. Thus, consumers’ decisions are influenced by peers (Hoonsopon and Puriwat, 2016).

Numerous studies have indicated that PI has an impact on consumer purchase intention. Samaraweera (2020) found that PI significantly reduced consumers’ purchase intention for crisis brands as PI played a moderating role in brand equity and purchase intention. Isa and Chin (2019) found that PI was positively associated with purchase intention when exploring the role of corporate social responsibility in ethical purchase intention. In summary, this article suggests that consumers’ final decision to purchase traceable food will be influenced by PI. If PI of traceable food is positive, the decrease in purchase intention caused by consumers’ original PR will be slightly slowed down. In contrast, the more positive the PI is, the more positive slope of PV on the purchase intention of traceable food will be inclined. Therefore, the following hypotheses were proposed:

**Hypothesis 9a**. PI moderates the negative relationship between PR and TFPI.

**Hypothesis 9b**. PI moderates the positive relationship between PV and TFPI.

Although the existing research results on consumers’ behavior are rich and diverse, there is still a lack of sufficient attention to the extrinsic cues of traceable food, and the research on consumers’ cognitive behavior of traceable food is just beginning. Specific types of research on identifying traceable information of interest to Chinese consumer are still limited (Liu et al., 2018). For example, current studies related to traceable food extrinsic cues are mostly related to tangible cues such as price and traceable information (Wu et al., 2016), while the role of intangible extrinsic cues such as consumers’ knowledge (Bai et al., 2013) and PI is neglected. Furthermore, there is limited research on consumers’ TFPI or the psychosocial prerequisites that influence these intentions (Menozzi et al., 2015). Research on the role of PV in traceability systems in the Chinese environment is not well documented (Lim et al., 2014). Meanwhile, although different scholars have conducted existential tests, identification, application, and other discussions around PI, peer research in the field of consumer behavior is still rare in China.

**FIGURE 1:**
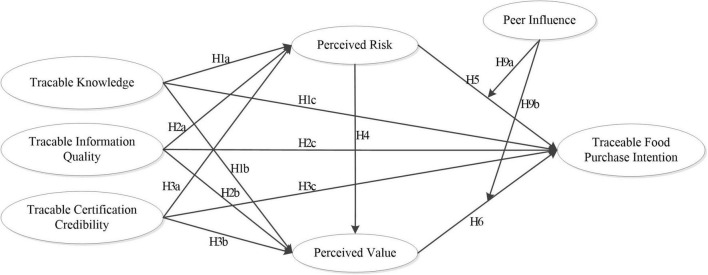
Research framework.

Based on the above study, this article proposes the research framework as shown in Figure 1. The study takes TK, TIQ, and TCC as independent variables; PR and PV as mediating variables; and purchase intention as dependent variable. It is also worth noting that this study considers PI in the social networking context as a moderating variable to explore its effect between cognitive evaluation and purchase intention.

## Materials and Methods

### Research Subjects and Data Collection

To empirically test the aforementioned model, this article designed a questionnaire based on the relevant constructs used in past literature and has been carefully verified by several scholars. A small-scale pretest of the questionnaire was conducted prior to large-scale distribution to test the reliability of the study. A total of 70 initial questionnaires were distributed to the investigators online and offline. A total of 63 questionnaires were returned and 7 invalid questionnaires were excluded, resulting in 56 pretest questionnaires with a return rate of 80%. After the preliminary partial least squares (PLS) algorithm, three questions did not meet the expected research objectives, namely, the outerloadings of TK3, TK4 (TK questions 3 and 4) and PR2 (PR question 2) were 0.676, 0.676, and 0.61, i.e., less than 0.7; thus, these three questions were deleted to improve the reliability and validity of the questionnaire. To ensure the quality and representativeness of the returned questionnaires, article conducted an online questionnaire survey and an offline questionnaire from July to September 2020. The online questionnaire survey was mainly placed on the website of Questionnaire Star. The offline questionnaire interview was mainly distributed to relatives, colleagues, friends, students, and their networks.

After 2 months of questionnaire distribution, 400 questionnaires were distributed online with 349 questionnaires collected. A total of 288 valid questionnaires were obtained after removing 61 invalid questionnaires. A total of 200 questionnaires were distributed offline with 157 questionnaires collected. A total of 133 valid questionnaires were obtained after removing 24 invalid questionnaires. A total of 600 questionnaires were distributed online and offline, 506 questionnaires were collected, and 421 valid questionnaires were obtained after deleting invalid questionnaires. The number of valid questionnaires/total distribution was 70%. According to Bagozzi and Yi (2012), the sample size should be at least 5 times the sample question items, and the ratio of the sample size to the sample question items in this paper is 12.38, which satisfies the sample size requirement.

### Measurement Instrument

Supported by a research project on “factors influencing consumers’ traceable food purchase intention,” this article is dedicated to analyzing and exploring the factors influencing the purchase of traceable food from consumers’ perspective, so as to help enterprises and upstream suppliers improve the accuracy of supply chain information flow, enhance the core competitiveness, and open up the market for traceable food. After a preliminary theoretical review, literature search, market research, and expert interviews, this article designed a three-part questionnaire.

The first part of the questionnaire mainly explained the source, basic information, and purpose of the survey to eliminate the concerns of the respondents in answering the questions and ensure the authenticity and reliability of the data collected as much as possible. The second part was the basic information of the survey respondents with a total of six items, including gender, education, age, income, purchase frequency, and purchase amount. The third part of the questionnaire focused on subjective measurement of seven dimensions (including TK, TIQ and TCC, PR, PV, TFPI, and PI). A total of 34 items were measured in this part, among which were 5 items of TK, drawing on the knowledge survey scale adopted by Wang and Tsai (2019) and Wang et al. (2018); 5 items of TIQ, drawing on the well-established scales of Yi et al. (2013) and Alma and Braimllari (2018); 5 items of TCC, drawing on the mature scales of Kim and Song (2020) and Zheng et al. (2017); 5 items of PR, drawing on the mature scales of Yi et al. (2013) and Alalwan et al. (2017); 5 items of PV, drawing on the mature scales of Alalwan et al. (2017) and Molinillo et al. (2021); 5 items of TFPI, drawing on the well-established scales of Kim and Song (2020) and Konuk (2018); and 4 items of PI, drawing on the well-established scales of Xia et al. (2012) and Isa and Chin (2019).

To improve the discrimination and increase the amount of variation between the latent variables, a 7-point Likert scale was adopted in this article. The latent variables were calculated ranging from “strongly disagree” (1) to “strongly agree” (7). Respondents rated each question in the range of 1–7 based on the description of the question and its degree of conformity to the actual situation, with higher scores representing greater agreement with the question.

### Research Method

In this article, SPSS 25.0 was used to perform sample descriptive statistics analysis, followed by a PLS path modeling approach using SmartPLS version 3.2.8, to validate and analyze the conceptual model. The main reasons for using PLS are as follows: (1) most marketing studies tend to have non-normal data assignment, while PLS does not require any assumption of normality and it handles non-normal assignment quite well and (2) PLS can overcome the multivariate covariance problem, effectively handle conditioning data and missing values, and has good predictive and explanatory power (Bontis et al., 2007).

The analysis and estimation procedure of PLS suggested by Vinzi et al. (2010) was adopted, which was divided into the following two phases: the first phase performed reliability and validity analysis, and the second phase performed path coefficients and explanatory power of the structural model. The two-phase analysis confirmed the reliability and validity of the constructs, thus verifying the relationships between constructs (Anderson and Gerbing, 1988).

## Results

### Descriptive Statistical Analysis

To test for possible non-response bias (NRB) and the representativeness of the sample, this article compared the earliest 10-day recalled samples (51) with the latest 10-day recalled samples (63), and conducted independent-sample *t*-tests for all latent variables in both subsamples. The results did not present statistically significant differences (Armstrong and Overton, 2005), representing that NRB did not have a serious impact in this article.

The results of the basic situation are detailed in Table 1. According to the statistical results, the gender ratio is consistent with the *status quo* that women are more active in food safety issues than men (Konuk, 2018). The results of the education level survey are also consistent with the *status quo* that users who purchase traceable food are generally highly educated. The monthly income structure basically conforms to the current status of the monthly income of urban residents in China, which also indicates that the current main consumer group’s monthly income is not high. It has also been found that the proportion of people who occasionally purchase traceable food is more than half, which is in line with the primary growth stage of the traceable food in the Chinese market. Furthermore, the results of purchase amount are also consistent with the miniaturization of traceable food purchase, that is, consumer are more inclined to make small and convenient multiple purchases, indicating that the consumers’ consumption has been changed from quantity demand to quality demand.

**TABLE 1:** Frequency distribution table.

Features	Classification	Frequency	Frequency (%)	Cumulative frequency (%)
Gender	Men	139	33.01	33.01
	Women	282	66.99	100
Age	Under 25 years old	228	54.16	54.16
	25–40 years old	85	20.19	74.35
	40–60 years old	78	18.53	92.88
	Over 60 years old	30	7.12	100
Education level	College and below	174	41.33	41.33
	Undergraduate	200	47.50	88.83
	Master and above	47	11.17	100
Monthly income	Under 3000 RMB	242	57.48	57.48
	3001–5000 RMB	71	16.87	74.35
	5001–8000 RMB	44	10.45	84.80
	More than 8000 RMB	64	15.20	100
Purchase frequency	Never	163	38.72	38.72
	Occasional purchases	227	53.92	92.64
	Frequent purchases	31	7.36	100
Purchase amount	Under 50 RMB	199	47.27	47.27
	50–100 RMB	112	26.61	73.88
	100–200 RMB	55	13.06	86.94
	Over 200 RMB	55	13.06	100

*n = 421.*

Variance inflation factor (VIF) can be used to ensure that there is no multicollinearity problem (Petter et al., 2007). Hair et al. (2012) suggested that when the VIF value is greater than or equal to 5, it implies the existence of a possible collinearity problem among the constructs. However, Table 2 shows that the VIF of all constructs is between 1.000 and 4.334, indicating that multicollinearity is not a problem in this research.

**TABLE 2:** Multicollinearity statistics.

Constructs	PI	PR	PV	TFPI
PI				
PR			3.027	4.334
PV				3.827
TCC		1.543	1.578	1.583
TFPI	1.000			
TIQ		2.682	3.791	3.926
TK		2.445	2.543	2.598

*TK, traceability knowledge; TIQ, traceable information quality; TCC, traceable certification credibility; PR, perceived risk; PV, perceived value; TFPI, traceability food purchase intention; PI, peer influence.*

### Outer Model and Scale Validation

The related tests for the outer model included the internal consistency, reliability, convergence validity, and discriminant validity to demonstrate the appropriateness of the constructs. Fornell and Larcker (1981) stated that the composite reliability (CR) and Cronbach’s α should be greater than 0.70, indicating the internal consistency of the items. Chin and Marcoulides (1998) suggested that the reliability can be judged by the factor loadings being greater than 0.7. AVE represents the average explanatory power of the constructs, whose value must be greater than 0.5 (Barclay et al., 1995). The confirmatory factor analysis (CFA) results show that the fact loadings, Cronbach’s α, and CR values of all constructs are greater than 0.70 and the AVE values are greater than 0.50 (Table 3), thus supporting the internal consistency, reliability, and convergence validity.

**TABLE 3:** Confirmatory factor analysis and scale reliability.

Constructs	Items	Loadings	Cronbach’s α	CR	AVE
TK	TK1	0.867	0.835	0.901	0.752
	TK2	0.882			
	TK5	0.853			
TIQ	TIQ1	0.860	0.900	0.926	0.714
	TIQ2	0.871			
	TIQ3	0.846			
	TIQ4	0.827			
	TIQ5	0.820			
TCC	TCC1	0.717	0.839	0.885	0.608
	TCC2	0.837			
	TCC3	0.733			
	TCC4	0.788			
	TCC5	0.816			
PR	PR1	0.837	0.886	0.921	0.745
	PR3	0.878			
	PR4	0.873			
	PR5	0.864			
PV	PV1	0.863	0.923	0.942	0.765
	PV2	0.863			
	PV3	0.895			
	PV4	0.877			
	PV5	0.873			
TFPI	TFPI1	0.882	0.887	0.918	0.692
	TFPI2	0.913			
	TFPI3	0.798			
	TFPI4	0.717			
	TFPI5	0.835			
PI	PI1	0.799	0.826	0.885	0.658
	PI2	0.822			
	PI3	0.863			
	PI4	0.756			

*TK, traceability knowledge; TIQ, traceable information quality; TCC, traceable certification credibility; PR, perceived risk; PV, perceived value; TFPI, traceability food purchase intention.*

Discriminant validity describes the degree of difference between a construct and other constructs. Fornell-Larcker criterion (Fornell and Larcker, 1981) is an important method to determine the discriminant validity. If a measurement model has discriminant validity, the square root of the AVE for each construct should be greater than the correlation coefficient between that construct and any other constructs (Hair et al., 2010). Table 4 shows the results of the discriminant validity. Except for the correlation coefficient between TFPI and PV (0.850), which is larger than the squared roots of AVE for TFPI (0.832). Any other AVE on the diagonal is larger than the correlation coefficient to its left and below, thus, TFPI and PV may have discriminant validity problem.

**TABLE 4:** Discriminant validity analysis.

Constructs	PI	PR	PV	TCC	TFPI	TIQ	TK
PI	**0.811**						
PR	0.735	**0.863**					
PV	0.773	0.840	**0.874**				
TCC	0.557	0.551	0.532	**0.780**			
TFPI	0.780	0.774	0.850	0.516	**0.832**		
TIQ	0.679	0.804	0.771	0.580	0.713	**0.845**	
TK	0.602	0.696	0.690	0.522	0.701	0.763	**0.867**

*TK, traceability knowledge; TIQ, traceable information quality; TCC, traceable certification credibility; PR, perceived risk; PV, perceived value; TFPI, traceability food purchase intention. The boldfaced diagonal elements are the square roots of AVE. Other elements are simple bivariate correlations between the constructs.*

To further examine the discriminant validity, the cross-loadings have been analyzed. As mentioned in Table 5, the factor loadings of each TFPI item are greater than its cross-loadings with PV; therefore, it can be concluded that the differential validity between these two variables should be valid at least within an acceptable range (Fornell and Larcker, 1981; Vinzi et al., 2010). In addition, one of the reasons why PLS is widely used is that its results are robust for multivariate covariance; in other words, the results of PLS will not be seriously affected by the lack of discriminant validity due to the high correlation between two variables (multivariate covariance). Although TFPI and PV in this study failed the Fornell-Larcker criterion, they should not cause serious distortion to the results of the subsequent statistical analysis.

**TABLE 5:** Summary table of cross-loading.

Title item	PI	PR	PV	TCC	TFPI	TIQ	TK
–	–	–	–	–	–	–	–
TFPI1	0.677	0.708	0.780	0.487	0.882	0.680	0.692
TFPI2	0.696	0.720	0.808	0.468	0.913	0.679	0.663
TFPI3	0.623	0.574	0.612	0.464	0.798	0.534	0.551
TFPI4	0.532	0.542	0.622	0.260	0.717	0.428	0.392
TFPI5	0.703	0.655	0.694	0.443	0.835	0.611	0.579
–	–	–	–	–	–	–	–

*TK, traceability knowledge; TIQ, traceable information quality; TCC, traceable certification credibility; PR, perceived risk; PV, perceived value; TFPI, traceability food purchase intention. – indicates data omission.*

### Inner Model and Hypotheses Testing

**FIGURE 2:**
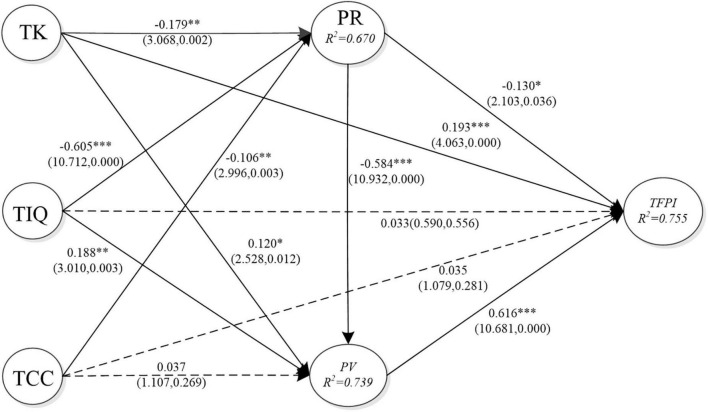
Standardized path coefficients and significance of the inner model.

Figure 2 shows the standardized path coefficient analysis. TK (β = −0.179, *p* = 0.002 < 0.01), TIQ (β = −0.605, *p* = 0.000 < 0.001), and TCC (TCC)(β = −0.106, *p* = 0.003 < 0.01) have a significant impact on PR; therefore, H1a, H2a, and H3a are supported. TK (β = 0.120, *p* = 0.012 < 0.05) and TIQ (β = 0.188, *p* = 0.003 < 0.01) have a significant impact on PV, while TCC (β = 0.037, *p* = 0.269 > 0.05) has no significant impact on PV; therefore, H1b and H2b are supported but H3b is not supported. TK (β = 0.193, *p* = 0.000 < 0.001) has a significant impact on TFPI, while TIQ (β = 0.33, *p* = 0.556 > 0.05) and TCC (β = 0.035, *p* = 0.281 > 0.05) have no significant impact on TFPI; therefore, H1c is supported but H2c and H3c are not supported. PR (β = −0.584, *p* = 0.000 < 0.001) has a significant impact on PV, supporting H4 of the research study. PV (β = 0.616, *p* = 0.000 < 0.001) and PR (β = −0.130, *p* = 0.036 < 0.05) have a significant impact on TFPI, supporting H5 and H6 of the research study.

The goal of PLS is to maximize the explained capacity of the endogenous variables. Like in multiple regressions, the *R*^2^ value is the portion of the total variance of a latent variable that can be explained. It is considered substantial if its value approximates 0.67, moderate if its value is around 0.33, and weak if its value is around 0.19 (Chin and Marcoulides, 1998). This article found that the degree of explained variation by PR and PV on TFPI was 75.5%. In contrast, TIQ, TK, and TCC have 67.0% of the explained variation for PR. Moreover, TCC, TIQ, and TK also had a high degree of variation of 73.9% for PV. The explanatory power of the relevant variables in this model is greater than or equal to 0.67, which means that the model has a fairly good explanatory power.

### Mediation Effect Analysis

Changes in the exogenous constructs lead to changes in the mediating variable, which in turn lead to changes in the endogenous constructs in the PLS path model. Mediating variables control the nature of the relationship between two constructs (i.e., the underlying mechanism or process). Understanding the issue of mediation is important in at least two ways as follows: (1) they are fundamental to explaining the management theme of how certain process factors can ameliorate or impede the impact of success drivers (Castro and Roldán, 2013) and (2) incorporating a third variable that plays an intermediate role between two variables in the model is a methodological challenge (Nitzl et al., 2016). Table 6 shows the result of mediating effect analysis. It can be found that H7b, H8a, and H8b are valid while H7a, H7c, and H8c are invalid.

**TABLE 6:** Mediating effect analysis.

Paths	Initial sample (O)	Standard Error (STERR)	T-statistic (|O/STERR|)	Significance (*P* < 0.05)
TK→PR→TFPI	0.023	0.014	1.673	0.094
TK→PV→TFPI	0.074	0.031	2.358	0.018*
TIQ→PR→TFPI	0.079	0.038	2.081	0.038*
TIQ→PV→TFPI	0.116	0.040	2.928	0.003**
TCC→PR→TFPI	0.014	0.008	1.699	0.089
TCC→PV→TFPI	0.023	0.021	1.089	0.276

TK, traceability knowledge; TIQ, traceable information quality; TCC, traceable certification credibility; PR, perceived risk; PV, perceived value; TFPI, traceability food purchase intention. *p-value < 0.05; **p-value < 0.01.

### Moderating Effect Analysis

Moderating effect refers to whether a variable can systematically change the form or strength of the correlation between the independent and dependent variables. In terms of statistical significance, this article intends to test whether different levels of PI make a significant difference in the effect between PV (PR) and purchase intention. To perform the moderating effect analysis, the built-in function of SmartPLS can easily multiply the independent variables with the indicator variables of the moderating variables and achieve the mean-center effect. Table 7 shows that the cross-term of PV and PI has a significant effect on TFPI (β = 0.021, *p* = 0.044 < 0.05), and the interaction of PR and PI has also a significant effect on TFPI (β = −0.026, *p* = 0.043 < 0.05); therefore, PI has a moderating effect both on the relationship between PV and TFPI, and between PR and TFPI. H9a and H9b are both valid.

**TABLE 7:** Moderating effect analysis.

Paths	Initial sample (O)	Standard Error (STERR)	T-statistic (|O/STERR|)	Significance (*P* < 0.05)
PR * PI→TFPI	–0.026	0.013	2.031	0.043*
PV * PI→TFPI	0.021	0.010	2.019	0.044*

*PR, perceived risk; PV, perceived value; TFPI, traceability food purchase intention.*

*p-value < 0.05.

## Discussion

The key to facilitate the benign development of the traceable food industry lies in improving the overall quality of traceable food products. This study attempts to explore the influence mechanism of TFPI from the perspective of consumers, and the influence and mechanism of the extrinsic cues of traceable food, consumers’ cognitive evaluation and PI in consumers’ purchase decision of traceable food have been clarified in order to provide implementable measures and suggestions for the traceable food industry, improve the quality of traceable food, assist enterprises to stay ahead in the industry competition, and boost the rapid development of the traceability industry.

## Conclusion

The findings show a significant relationship between TK and consumer’s cognitive evaluation, which are consistent with the findings of Wang et al. (2018) and Lin et al. (2017). This article argues that due to the large amount of detailed information carried by traceability labels, the information asymmetry problem in the food supply chain has been reduced effectively. The more consumers know about traceable foods, the more they can perceive this reduction in food safety risk, and the increase in value through traceability labels. The empirical results show that TIQ has a significant effect on PR and PV, which are consistent with the studies of Mun et al. (2013) and Zheng et al. (2017). The results suggest that detailed information can provide predictive food quality. If the traceable information is perceived as a valuable source of information, high information quality is more likely to motivate consumers to affirm the value of traceable food and reduce their concerns about its risks. The empirical results between TCC and consumers’ cognitive evaluation show that the traceable credibility obtained from certification marks has a significant negative effect on PR, which is also confirmed in a previous study by Brach et al. (2017). In contrast, the influence of TCC on PV was not significant, indicating that the certification mark significantly reduced consumers’ PR in their food purchase and alleviated their concerns about food safety risks, but did not cause a change in their PV. Possible explanations are, although China has established national standards and industry standards for traceable foods, there are still problems such as the lack of detailed descriptions of food cultivation, distribution and sales, the low information content of traceability codes, the cumbersome inquiry process, and the lack of credible certification, all of which lead to information collection costs exceeding marginal benefits for consumers, resulting in consumers choosing foods that are less risky but familiar. Certification marks act as an implication of quality, conveying attributes such as sustainability and healthiness (Grunert and Aachmann, 2016), and have been increasingly applied in association with products or brands with social value (Brach et al., 2017).

The empirical results of the direct relationship between extrinsic cues of traceable food and TFPI indicate that TK enhances consumers’ cognitive evaluation (Wang and Hazen, 2015) and purchase intention (Wang and Tsai, 2019), thus confirming that knowledge is an important dimension in forming cognitive evaluation and behavioral intention (Jorgensen and Savla, 2010). However, the positive effects of TIQ and TCC on purchase intention were not significant. This may be due to the current food sampling system in China, which makes consumers believe that the food quality represented by food labels is not sufficient to make their purchase decision; meanwhile, the certification mark does not release the credibility signal it represents, and is not yet sufficient to support consumers’ judgments about traceable food purchase decisions.

The results of the study show that PR has a significant negative impact on PV, and both have a significant effect on TFPI. Similar research was conducted by Tzavlopoulos et al. (2019). It indicates that PR and PV, representing components of consumers’ cognitive evaluation, reflect the psychological process of consumers’ purchase decision-making. Unlike other types of consumption, the reduction of risk associated with traceable food precedes the increase in value. As food consumers, they desire to minimize “food safety risks” while ensuring their purchase decisions are “informed” and provide them with long-term “health benefits, social status” and other values. As a result, they perceive risk ahead of value and attempt to remain rational in their purchase decisions to maximize benefits. Furthermore, according to the path coefficients, the influence of PV is stronger than that of PR, indicating that PV is a more important factor in the formation of consumers’ purchase intention. It validates Ponte et al.’s view that PV is a driving factor for consumers’ purchase behavior (Ponte et al., 2015). The research finding provides some insight into describing the types of traceable food consumers, while also enriching and complementing the existing research findings.

Economic theories such as prospect theory (Tversky, 1979) suggest that consumers usually seek to evaluate value and risk as a whole when making purchase decisions. The empirical results suggest that it is the same for consumers’ decision-making on traceable food. The results of the mediation effects show that PV and PR are two important mediating variables in this research model, which play different mediating roles in the mechanisms of TK and TIQ on TFPI.

Taking PI as a breakthrough point, this research investigates the role played by PI between cognitive evaluation and TFPI after consumers formed their own cognitive evaluation. In the turbulent Internet age, peer has the potential to influence consumers’ established evaluation of traceable food purchase. In other words, the influence of the consumers’ own cognitive evaluation on purchase intention is weakened when the PI is high enough. This result allows for a more nuanced interpretation of the relationship between consumers’ cognitive evaluation and purchase intention, and also expands on existing PI and PV research. For example, some studies have shown a direct positive relationship between PI and consumers’ PV (Lin and Bautista, 2020), while the results of some others are irrelevant (Belz and Schmidt-Riediger, 2010). This study expands the research in this area by clarifying the role of PI in the Chinese context and giving an alternative way of PI.

### Implications

This research constructs a conceptual model of extrinsic cues of traceable food and TFPI to reveal the logic of “cue-cognition-decision” and provides research support to explain consumers’ “cognition-intention” gap.

The research provides strong evidence for the view that TK is a very important antecedent factor in consumers’ decision-making process (Wang and Hazen, 2015). The results showed that the intangible extrinsic cue of TK has a more intricate mechanism of influence on purchase intention, and the cultivation of knowledge requires long-term accumulation; therefore, as a sustainable industry, the promotion of traceable food requires not only short-term publicity but also long-term accumulation of knowledge and experience. The concern for food safety and traceable food should start from education, shaping the concept of traceability from childhood, improving TK literacy, and using knowledge to drive and influence more consumers.

Due to the positive relationship between traceability information quality and PR and PV, scholars have called for a shift in business models from managing customer to value creation and value chain development (Tretyak et al., 2013), where companies can make changes through product traceability, information, and service upgrades that affect resources, processes, products, services, and solutions along the supply chain (Kohtamaki and Rajala, 2016). The importance of traceability information quality is highlighted by improving the quality of traceability code information as a way to ensure the increasingly intuitive appeal of traceable food and consumers’ positive purchase intention.

The empirical results showed that consumers have doubts about TCC. The contrast between the important role of the certification mark and the empirical results precisely indicates that it is crucial to develop high-quality standards for traceable food and provide consumers with certification of these standards (Giacomarra et al., 2016). In the turbulent Internet age, it is important to develop a risk-averse mechanism for the government and industry, and there is an urgent need for the government, non-profit organizations, research institutions, or academic units to take the lead in organizing and implementing third-party certification of traceable food, achieve the management and supervision of enterprises, release the transmission of trust represented by certification to the market, and convey reliable and accurate information of traceable food to the consumers.

The moderating role of peers suggests that traceable food companies should pay attention to the social environment where traceable food is sold. By building an exclusive social interaction platform, the company cultivates lead users and members who can grasp public opinion and be more effective or influential, so as to attract more consumer; and by the consumption of high-quality traceable food, the company brings about members’ self-fulfillment, enhancement, and satisfaction of membership status and social status, thus realizing the cultivation of consumption habit and loyalty of members as well as the promotion of traceable food market positioning. Eventually, through the product differentiation of “traceability code and certification mark,” “get what one pays for” can be realized and a virtuous cycle of high quality brought by high selling price can be formed.

In summary, as a food with higher technological content, buy-back behavior is what sustainable food industry expects and cares about. This research attempts to explain the decision-making process and consumer behavior of traceable food from the initial purchase intention to the final purchase experience, with the hope that the research can be applied to any scenario of future research with a more profound way of thinking. The study concluded that when implementing precision marketing, companies should not only focus on short-term advertising campaigns but should also take root downward and start from education to cultivate the concept of traceable consumption. At the same time, although the quality of traceable food is the top priority, it is also crucial to pay attention to communication and interaction with consumer.

### Research Limitations and Future Research Suggestions

Although the intended research objectives of this article were met, there were several limitations that can be addressed for future research. First, due to the geographical and time constraints, there may be inevitably groups not covered by the questionnaire. Respondents’ answers may also be influenced by subjective reasons (social expectations, subjective feelings, attitudes, emotions, etc.), resulting in less objective questionnaire results. In the future, the cooperation with publishing associations and publishers can be considered to expand the group coverage and sample size in order to increase the breadth of this research and the rigor of the findings through randomization and sampling considerations. Second, this article investigated the relationship between extrinsic cues of traceable food and purchase intention from the perspective of consumers’ advanced cognition, while in reality, consumers’ cognitive evaluation is a gradual process of “primary cognition-emotion-advanced cognition,” thus more factors should be analyzed in future research. For example, more sociodemographic factors (family size, children, health status, etc.) or emotional factors (satisfaction, loyalty, etc.) in the cognitive-psychological structure should be included in future research to further analyze the interaction between different influencing factors, increase the explanatory power of the model, and promote the further development of the study. Finally, although this article provides important preliminary insights into the influence of extrinsic cues of traceable food on consumers’ purchase intention, additional field studies are needed to confirm the validity of the results. As the traceability system is still in improvement, and industrial application has not yet been popularized, research on the purchase intention of traceable food is limited. Most of the relevant studies are single-issue studies; comprehensive information attributes of traceable food and the influence of traceability certification credibility bodies are rare. Therefore, it is difficult to corroborate the results by typical cases. In the future, the reasonableness of the model will be verified through more cases and research results.

## Data Availability Statement

The raw data supporting the conclusions of this article will be made available by the author, without undue reservation.

## Author Contributions

LG contributed to the conceptualization, original draft preparation, review and editing, and visualization. The author has read and agreed to the published version of the manuscript.

## Conflict of Interest

The author declares that the research was conducted in the absence of any commercial or financial relationships that could be construed as a potential conflict of interest.

## Publisher’s Note

All claims expressed in this article are solely those of the authors and do not necessarily represent those of their affiliated organizations, or those of the publisher, the editors and the reviewers. Any product that may be evaluated in this article, or claim that may be made by its manufacturer, is not guaranteed or endorsed by the publisher.

## References

[B1] Abror A. Patrisia D. Engriani Y. Omar M. W. Najib M. (2021). Perceived risk and tourist’s trust: the roles of perceived value and religiosity. *J. Islam. Mark.* [Epub ahead-of-print], 10.1108/JIMA-03-2021-0094

[B2] Alalwan A. A. Dwivedi Y. K. Rana N. P. Algharabat R. (2017). Examining factors influencing Jordanian customers’ intentions and adoption of internet banking: extending utaut2 with risk. *J. Retail. Consum. Serv.* 40 125–138. 10.1016/j.jretconser.2017.08.026

[B3] Alma S. Braimllari S. A. (2018). Information technology inhibitors and information quality in supply chain management: a pls-sem analysis. *Acad. J. Interdiscip. Stud.* 7:125. 10.2478/ajis-2018-0064

[B4] Anderson J. C. Gerbing W. (1988). Structural equation modeling in practice: a review and recommended two-step approach. *Psychol. Bull.* 27 5–24. 10.1037//0033-2909.103.3.411

[B5] Armstrong J. S. Overton T. S. (2005). Estimating nonresponse bias in mail survey. *J. Mark. Res.* 14 396–402. 10.1177/002224377701400320

[B6] Arnold M. B. (2010). Emotion, motivation, and the limbic system. *Ann. N. Y. Acad. Sci.* 159 1041–1058. 10.1111/j.1749-6632.1969.tb12996.x 4900682

[B7] Badia-Melis R. Mishra P. Ruiz-García L. (2015). Food traceability: new trends and recent advances. A review. *Food Control* 57 393–401. 10.1016/j.foodcont.2015.05.005

[B8] Bagozzi R. P. Yi Y. (2012). Specifications, evaluation, and interpretation of structural equation models. *J. Acad. Mark. Sci.* 40 8–34. 10.1007/s11747-011-0278-x

[B9] Bai J. Zhang C. Jiang J. (2013). The role of certificate issuer on consumers’ willingness-to-pay for milk traceability in China. *Agric. Econ.* 44 537–544. 10.1111/agec.12037

[B10] Barclay D. Higgins C. Thomson R. (1995). The partial last squares (PLS) approach to causal modelling, personal computer adoption and use as an illustration. *Technol. Stud.* 2 285–309.

[B11] Belz F. M. Schmidt-Riediger B. (2010). Marketing strategies in the age of sustainable development: evidence from the food industry. *Bus. Strategy Environ.* 19 401–416. 10.1002/bse.649

[B12] Bernués A. Olaizola A. Corcoran K. (2003). Extrinsic attributes of red meat as indicators of quality in Europe: an application for market segmentation. *Food Qual. Prefer.* 14 265–276. 10.1016/S0950-3293(02)00085-X

[B13] Bontis N. Booker L. D. Serenko A. (2007). The mediating effect of organizational reputation on customer loyalty and service recommendation in the banking industry. *Manag. Decis.* 45 1426–1445. 10.1108/00251740710828681

[B14] Brach S. Walsh G. Shaw D. (2017). Sustainable consumption and third-party certification labels: consumers’ perceptions and reactions. *Eur. Manag. J. EMJ* 36 254–265. 10.1016/j.emj.2017.03.005

[B15] Buaprommee N. Polyorat K. (2016). The antecedents of purchase intention of meat with traceability in thai consumers. *Asia Pac. Manag. Rev.* 21 161–169. 10.1016/j.apmrv.2016.03.001

[B16] Campbell E. Q. Alexander C. N. (1965). Structural effects and interpersonal relationships. *Am. J. Sociol.* 71 284–289. 10.1086/224087 5897474

[B17] Castro I. Roldán J. L. (2013). A mediation model between dimensions of social capital. *Int. Bus. Rev.* 22 1034–1050. 10.1016/j.ibusrev.2013.02.004

[B18] Cheung C. Lee M. Rabjohn N. (2008). The impact of electronic word-of-mouth – the adoption of online opinions in online customer communities. *Internet Res.* 18 229–247. 10.1108/10662240810883290

[B19] Chin W. W. Marcoulides G. (1998). The partial least squares approach to structural equation modeling. *Adv. Hosp. Leis.* 8 295–336.

[B20] Cox D. F. (1962). The measurement of information value: a study in consumer decision -making. *Emerg. Concepts Mark.* 1962 413–421.

[B21] Cui L. Jiang H. Deng H. Zhang T. (2019). The influence of the diffusion of food safety information through social media on consumers’ purchase intentions. *Data Technol. Appl.* 53 230–248. 10.1108/DTA-05-2018-0046

[B22] Das G. (2015). Linkages between self-congruity, brand familiarity, perceived quality and purchase intention: a study of fashion retail brands. *J. Glob. Fashion Mark.* 6 180–193. 10.1080/20932685.2015.1032316

[B23] Filieri R. (2015). What makes online reviews helpful? A diagnosticity-adoption framework to explain informational and normative influences in e-WOM. *J. Bus. Res.* 68, 1261–1270. 10.1016/j.jbusres.2014.11.006

[B24] Fornell C. Larcker D. F. (1981). Structural equation models with unobservable variables and measurement error: algebra and statistics. *J. Mark. Res.* 18 382–388. 10.1177/002224378101800313

[B25] Giacomarra M. Galati A. Crescimanno M. Tinervia S. (2016). The integration of quality and safety concerns in the wine industry: the role of third-party voluntary certifications. *J. Clean. Prod.* 112(Pt 1) 267–274. 10.1016/j.jclepro.2015.09.026

[B26] Grunert K. G. Aachmann K. (2016). Consumer reactions to the use of EU quality labels on food products: a review of the literature. *Food Control* 59 178–187. 10.1016/j.foodcont.2015.05.021

[B27] Hair J. F. Black W. C. Babin B. J. Anderson R. E. (2010). *Multivariate Data Analysis: A Global Perspective*, 7th Edn. Upper Saddle River, NJ: Pearson Prentice Hall.

[B28] Hair J. F. Ringle C. M. Sarstedt M. (2012). Partial least squares: the better approach to structural equation modeling? *Long Range Plann.* 45 312–319. 10.1016/j.lrp.2012.09.011

[B29] Hallinan M. T. Williams R. A. (1990). Students’ characteristics and the peerinfluence process. *Sociol. Educ.* 63 122–132. 10.2307/2112858

[B30] Hamlin R. P. (2010). Cue-based decision making. A new framework for understanding the uninvolved food consumer. *Appetite* 55 89–98. 10.1016/j.appet.2010.04.007 20420871

[B31] Han M. C. Kim Y. (2016). Why consumers hesitate to shop online: perceived risk and product involvement on taobao.com. *J. Promot. Manag.* 23 1–21. 10.1080/10496491.2016.1251530

[B32] Hobbs J. E. (2004). Information asymmetry and the role of traceability systems. *Agribusiness* 20 397–415. 10.1002/agr.20020

[B33] Hoonsopon D. Puriwat W. (2016). The effect of reference groups on purchase intention: evidence in distinct types of shoppers and product involvement. *Australas. Mark. J.* 24 157–164. 10.1016/j.ausmj.2016.05.001

[B34] Hsu C. L. Chang K. C. Chen M. C. (2012). The impact of website quality on customer satisfaction and purchase intention: perceived playfulness and perceived flow as mediators. *Inform. Syst. eBus. Manag.* 10 549–570. 10.1007/s10257-011-0181-5

[B35] Isa S. M. Chin P. N. (2019). Exploring the role of corporate social responsibility skepticism in ethical purchase intention. *Soc. Responsib. J.* 16 291–307. 10.1108/SRJ-01-2018-0003

[B36] Jayashankar P. Nilakanta S. Johnston W. J. Gill P. Burres R. (2018). Iot adoption in agriculture: the role of trust, perceived value and risk. *J. Bus. Ind. Mark.* 33 804–821. 10.1108/JBIM-01-2018-0023

[B37] Jorgensen B. L. Savla J. (2010). Financial literacy of young adults: the importance of parental socialization. *Fam. Relat.* 59 465–478. 10.1111/j.1741-3729.2010.00616.x

[B38] Kandel D. B. (1985). On processes of peer influences in adolescent drug use: a developmental perspective. *Adv. Alcohol Subst. Abuse* 4 139–163. 10.1300/J251v04n03_07 3874527

[B39] Kim J. H. Song H. (2020). The influence of perceived credibility on purchase intention via competence and authenticity. *Int. J. Hosp. Manag.* 90:102617. 10.1016/j.ijhm.2020.102617

[B40] Kim W. G. Moon Y. J. (2009). Customers’ cognitive, emotional, and actionable response to the servicescape: a test of the moderating effect of the restaurant type. *Int. J. Hosp. Manag.* 28 144–156. 10.1016/j.ijhm.2008.06.010

[B41] Klerck D. Sweeney J. C. (2010). The effect of knowledge types on consumer-perceived risk and adoption of genetically modified foods. *Biotechnol. J.* 24 171–193. 10.1002/mar.20157

[B42] Kohtamaki M. Rajala R. (2016). Theory and practice of value co-creation in b2b systems. *Ind. Mark. Manag.* 56 4–13. 10.1016/j.indmarman.2016.05.027

[B43] Konuk F. A. (2018). The role of store image, perceived quality, trust and perceived value in predicting consumers’ purchase intentions towards organic private label food. *J. Retail. Consum. Serv.* 43 304–310. 10.1016/j.jretconser.2018.04.011

[B44] Liang L. J. Choi H. C. Joppe M. (2015). Understanding repurchase intention of airbnb consumers: perceived authenticity, electronic word-of-mouth, and price sensitivity. *J. Travel Tour. Mark.* 35 1–17. 10.1080/10548408.2016.1224750

[B45] Lim W. M. Yong J. L. S. Suryadi K. (2014). Consumers’ perceived value and willingness to purchase organic food. *J. Glob. Mark.* 27 298–307. 10.1080/08911762.2014.931501

[B46] Lin J. Lobo A. Leckie C. (2017). The influence of green brand innovativeness and value perception on brand loyalty: the moderating role of green knowledge. *J. Strateg. Mark.* 27 1–15. 10.1080/0965254X.2017.1384044

[B47] Lin T. T. C. Bautista J. R. (2020). Content-related factors influence perceived value of location-based mobile advertising. *J. Comput. Inform. Syst.* 60, 184–193. 10.1080/08874417.2018.1432995

[B48] Liu A. Niyongira R. (2017). Chinese consumers food purchasing behaviors and awareness of food safety. *Food Control* 79 185–191. 10.1016/j.foodcont.2017.03.038

[B49] Liu C. Bao Z. Zheng C. (2019). Exploring consumers’ purchase intention in social commerce: an empirical study based on trust, argument quality, and social presence. *Asia Pac. J. Mark. Logist.* 31 378–397. 10.1108/APJML-05-2018-0170

[B50] Liu C. Li J. Steele W. Fang X. Nath H. K. (2018). A study on Chinese consumer preferences for food traceability information using best-worst scaling. *PLoS One* 13:e0206793. 10.1371/journal.pone.0206793 30388166 PMC6214548

[B51] McClure C. Seock Y. K. (2020). The role of involvement: investigating the effect of brand’s social media pages on consumer purchase intention. *J. Retail. Consum. Serv.* 53:101975. 10.1016/j.jretconser.2019.101975

[B52] Menozzi D. Halawany-Darson R. Mora C. Giraud G. (2015). Motives towards traceable food choice: a comparison between French and Italian consumers. *Food Control* 49 40–48. 10.1016/j.foodcont.2013.09.006

[B53] Mitchell V. W. (1999). Consumer perceived risk: conceptualizations and models. *Eur. J. Mark.* 33 163–195. 10.1108/03090569910249229

[B54] Molinillo S. Aguilar-Illescas R. Anaya-Sánchez R. Liébana-Cabanillas F. (2021). Social commerce website design, perceived value and loyalty behavior intentions: the moderating roles of gender, age and frequency of use. *J. Retail. Consum. Serv.* 63:102404. 10.1016/j.jretconser.2020.102404

[B55] Mun Y. Y. Yoon J. J. Davis J. M. Lee T. (2013). Untangling the antecedents of initial trust in web-based health information: the roles of argument quality, source expertise, and user perceptions of information quality and risk. *Decis. Support Syst.* 55 284–295. 10.1016/j.dss.2013.01.029

[B56] Nitzl C. Roldàn J. L. Carrión G. C. (2016). Mediation analysis in partial least squares path modeling: helping researchers discuss more sophisticated models. *Ind. Manag. Data Syst.* 116 1849–1864. 10.1108/IMDS-07-2015-0302

[B57] Olson J. C. Jacoby J. (1972). “Cue utilization in the quality perception process,” in *Proceedings of the 3rd Annual Conference of the Association for Consumer Research*, Chicago, IL, 167–179.

[B58] Paul A. J. Modi A. A. Patel J. B. (2016). Predicting green product consumption using theory of planned behavior and reasoned action. *J. Retail. Consum. Serv.* 29 123–134. 10.1016/j.jretconser.2015.11.006

[B59] Peng N. Chen A. (2019). Luxury hotels going green-the antecedents and consequences of consumer hesitation. *J. Sustain. Tour.* 27 1374–1392. 10.1080/09669582.2019.1622710

[B60] Petraviiūt P. Einauskiené B. Rūtelion A. Krukowski K. (2021). Linking luxury brand perceived value, brand attachment, and purchase intention: the role of consumer vanity. *Sustainability* 13:6912. 10.3390/su13126912

[B61] Petter S. Straub D. W. Rai A. (2007). Specifying formative constructs in information systems research. *MIS Q.* 31 623–656. 10.2307/25148814

[B62] Ponte E. B. Carvajal-Trujillo E. Escobar-Rodriguez T. (2015). Influence of trust and perceived value on the intention to purchase travel online: integrating the effects of assurance on trust antecedents. *Tour. Manag.* 47 286–302. 10.1016/j.tourman.2014.10.009

[B63] Riccioli F. Moruzzo R. Zhang Z. Zhao J. Guidi A. (2019). Willingness to pay in main cities of Zheijiang provice (China) for quality and safety in food market. *Food Control* 108:106831. 10.1016/j.foodcont.2019.106831

[B64] Richardson P. S. Dick A. S. Jain A. K. (1994). Extrinsic and intrinsic cue effects on perceptions of store brand quality. *J. Mark.* 58 28–36. 10.2307/1251914

[B65] Rijswijk W. V. Frewer L. J. Menozzi D. Faioli G. (2008). Consumer perceptions of traceability: a cross-national comparison of the associated benefits. *Food Qual. Prefer.* 19 452–464. 10.1016/j.foodqual.2008.02.001

[B66] Rogers S. Wheeler T. J. (1976). A study of the views of teachers of first year infant school children concerning the effects on the language and socialisation of children who have previously attended playgroups. *Aggression* 14 1–14.

[B67] Samaraweera G. C. (2020). “Effect of consumer based brand equity on purchase intention of the crisis brand: moderating role of peer recommendation,” in *Proceedings of the 6th International Symposium on Multidisciplinary Research for Sustainable Development in the Information Era*, Oluvil.

[B68] Schmidt W. E. Tyler V. O. (1975). The “pinpointing effect” vs. the “diffusion effect” of peer influence. *Psychol. Sch.* 12 484–494. 10.1002/1520-6807(197510)12:4<484::aid-pits2310120419<3.0.co;2-o

[B69] Shao B. J. Cheng Z. D. Wan L. J. Yue J. (2021). The impact of cross border e-tailer’s return policy on consumers’ purchase intention. *J. Retail. Consum. Serv.* 59:102367. 10.1016/j.jretconser.2020.102367

[B70] Sharaf M. A. Isa F. M. (2017). Factors influencing students’ intention to purchase green products: a case study in Universiti Utara Malaysia. *Pertanika J. Soc. Sci. Humanit.* 25 239–250.

[B71] SJTU Food Safety Research Center (2019). *Outbreak-Related Hard-Boiled Egg Recall Now Includes Consumer Products.* Available online at: http://www.foodluh.sjtu.edu.cn/en/newsexpress/1243 (accessed January 15, 2021).

[B72] Suki N. M. (2016). Green product purchase intention: impact of green brands, attitude, and knowledge. *Br. Food J.* 118 2893–2910. 10.1108/BFJ-06-2016-0295

[B73] Sweeney J. C. Soutar G. N. Johnson L. W. (1999). The role of perceived risk in the quality-value relationship: a study in a retail environment. *J. Retail.* 75 77–105. 10.1016/S0022-4359(99)80005-0

[B74] Tran V. D. (2020). The relationship among product risk, perceived satisfaction and purchase intentions for online shopping. *J. Asian Financ. Econ. Bus.* 7 221–231. 10.13106/jafeb.2020.vol7.no6.221

[B75] Tretyak O. Tretyak O. A. Sloev I. (2013). Customer flow: evaluating the long-term impact of marketing on value creation. *J. Bus. Ind. Mark.* 28 221–228. 10.1108/08858621311302877

[B76] Tversky K. A. (1979). Prospect theory: an analysis of decision under risk. *Econometrica* 47 263–291. 10.2307/1914185

[B77] Tzavlopoulos I. Gotzamani K. Andronikidis A. Vassiliadis C. (2019). Determining the impact of e-commerce quality on customers’ perceived risk, satisfaction, value and loyalty. *Int. J. Qual. Serv. Sci.* 11 576–587. 10.1108/IJQSS-03-2019-0047

[B78] Ueland Ø Gunnlaugsdottir H. Holm F. Kalogeras N. Leino O. Luteijn J. M. (2012). State of the art in benefit–risk analysis: consumer perception. *Food Chem. Toxicol.* 50 67–76. 10.1016/j.fct.2011.06.006 21683114

[B79] Verbeke W. Ward R. W. (2006). Consumer interest in information cues denoting quality, traceability and origin: an application of ordered probit models to beef labels. *Food Qual. Prefer.* 17 453–467. 10.1016/j.foodqual.2005.05.010

[B80] Vinzi V. E. Henseler J. Chin W. W. (2010). *Handbook of Partial Least Squares: Concepts, Methods and Applications.* Berlin: Springer-Verlag.

[B81] Wang J. Yue H. Zhou Z. (2017). An improved traceability system for food quality assurance and evaluation based on fuzzy classification and neural network. *Food Control* 79 363–370. 10.1016/j.foodcont.2017.04.013

[B82] Wang S. Jing W. Li J. Wang J. Liang L. (2018). Policy implications for promoting the adoption of electric vehicles: do consumers’ knowledge, perceived risk and financial incentive policy matter? *Transp. Res. A Policy Pract.* 117 58–69. 10.1016/j.tra.2018.08.014

[B83] Wang S. T. Tsai M. C. (2019). Effects of the perception of traceable fresh food safety and nutrition on perceived health benefits, affective commitment, and repurchase intention. *Food Qual. Prefer.* 78:103723. 10.1016/j.foodqual.2019.103723

[B84] Wang Y. Hazen B. T. (2015). Consumer product knowledge and intention to purchase remanufactured products. *Int. J. Prod. Econ.* 181 460–469. 10.1016/j.ijpe.2015.08.031

[B85] Wu L. Wang H. Zhu D. Hu W. Wang S. (2016). Chinese consumers’ willingness to pay for pork traceability information—the case of wuxi. *Agric. Econ.* 47 71–79. 10.1111/agec.12210

[B86] Wu L. Wang S. Zhu D. Hu W. Wang H. (2015). Chinese consumers’ preferences and willingness to pay for traceable food quality and safety attributes: the case of pork. *China Econ. Rev.* 35 121–136. 10.1016/j.chieco.2015.07.001

[B87] Xia W. Yu C. Wei Y. (2012). Social media peer communication and impacts on purchase intentions: a consumer socialization framework. *J. Interact. Mark.* 26 198–208. 10.1016/j.intmar.2011.11.004

[B88] Yi M. Y. Yoon J. J. Davis J. M. Lee T. (2013). Untangling the antecedents of initial trust in web-based health information: the roles of argument quality, source expertise, and user perceptions of information quality and risk. *Decis. Support Syst.* 55 284–295.

[B89] Yoo C. W. Parameswaran S. Kishore R. (2015). Knowing about your food from the farm to the table: using information systems that reduce information asymmetry and health risks in retail contexts. *Inform. Manag.* 52 692–709. 10.1016/j.im.2015.06.003

[B90] Yuan C. Wang S. Yu X. (2020). The impact of food traceability system on consumer perceived value and purchase intention in china. *Ind. Manag. Data Syst.* 120 810–824. 10.1108/IMDS-09-2019-0469

[B91] Yue L. Liu Y. Wei X. (2017). Influence of online product presentation on consumers’ trust in organic food: a mediated moderation model. *Br. Food J.* 119 2724–2739. 10.1108/BFJ-09-2016-0421

[B92] Zeithaml V. A. (1988). Consumer perceptions of price, quality, and value: a means-end model and synthesis of evidence. *J. Mark.* 52 2–22. 10.2307/1251446

[B93] Zheng C. Yu X. Jin Q. (2017). How user relationships affect user perceived value propositions of enterprises on social commerce platforms. *Inform. Syst. Front.* 19 1–11. 10.1007/s10796-017-9766-y

